# Association between Blood Glucose and cardiac Rhythms during pre-hospital care of Trauma Patients – a retrospective Analysis

**DOI:** 10.1186/s13049-018-0516-z

**Published:** 2018-07-13

**Authors:** Janett Kreutziger, Stefan Schmid, Nikolaus Umlauf, Hanno Ulmer, Maarten W. Nijsten, Daniel Werner, Thomas Schlechtriemen, Wolfgang Lederer

**Affiliations:** 1 Department of Anaesthesia and Intensive Care Medicine, Medical University of Innsbruck, Anichstrasse 35, 6020 Innsbruck, Austria; 2 Department of General and Surgical Intensive Care Medicine, Medical University of Innsbruck, Anichstrasse 35, 6020 Innsbruck, Austria; 3 Department of Statistics, Faculty of Economics and Statistics, University of Innsbruck, Universitätsstrasse 15, 6020 Innsbruck, Austria; 4 Department of Medical Statistics, Informatics and Health Economics, Medical University of Innsbruck, Schöpfstrasse 41/1, 6020 Innsbruck, Austria; 5 University of Groningen, University Medical Centre Groningen, Hanzeplein 1, 9713 Groningen, GZ Netherlands; 6 German Helicopter Emergency Medical Services (ADAC Luftrettung gGmbH), Hansastrasse 19, 80686 Munich, Germany; 7 Emergency Medical Services of the Saarland, Saarpfalz-Park 9, 66450 Bexbach, Germany; 8 Formerly: Quality Management of the German Helicopter Emergency Medical Services (ADAC Luftrettung gGmbH), Hansastrasse 19, 80686 Munich, Germany; 9 Department of Anaesthesia and Intensive Care Medicine, Medical University of Innsbruck, Anichstrasse 35, 6020 Innsbruck, Austria

**Keywords:** Trauma, Cardiac arrest, Tachyarrhythmia, Bradyarrhythmia, Pre-hospital care, Blood glucose

## Abstract

**Background:**

Deranged glucose metabolism is frequently observed in trauma patients after moderate to severe traumatic injury, but little data is available about pre-hospital blood glucose and its association with various cardiac rhythms and cardiac arrest following trauma.

**Methods:**

We retrospectively investigated adult trauma patients treated by a nationwide helicopter emergency medical service (34 bases) between 2005 and 2013. All patients with recorded initial cardiac rhythms and blood glucose levels were enrolled. Blood glucose concentrations were categorised; descriptive and regression analyses were performed.

**Results:**

In total, 18,879 patients were included, of whom 185 (1.0%) patients died on scene. Patients with tachycardia (≥100/min, 7.0 ± 2.4 mmol/L *p* < 0.0001), pulseless ventricular tachycardia (9.8 ± 1.8, mmol/L, *p* = 0.008) and those with ventricular fibrillation (9.0 ± 3.2 mmol/L, p < 0.0001) had significantly higher blood glucose concentrations than did patients with normal sinus rhythm between 61 and 99/min (6.7 ± 2.1 mmol/L). In patients with low (≤2.8 mmol/L, 7/79; 8.9%, p < 0.0001) and high (> 10.0 mmol/L, 70/1271; 5.5%, p < 0.0001) blood glucose concentrations cardiac arrest was more common than in normoglycaemic patients (166/9433, 1.8%). ROSC was more frequently achieved in hyperglycaemic (> 10 mmol/L; 47/69; 68.1%) than in hypoglycaemic (≤4.2 mmol/L; 13/31; 41.9%) trauma patients (*p* = 0.01).

**Conclusions:**

In adult trauma patients, pre-hospital higher blood glucose levels were related to tachycardic and shockable rhythms. Cardiac arrest was more frequently observed in hypoglycaemic and hyperglycaemic pre-hospital trauma patients. The rate of ROSC rose significantly with rising blood glucose concentration. Blood glucose measurements in addition to common vital parameters (GCS, heart rate, blood pressure, breathing frequency) may help identify patients at risk for cardiopulmonary arrest and dysrhythmias.

## Background

In-hospital hypo- and hyperglycaemia are known to be predictive for outcome in several acute and critical diseases [1–3], but especially trauma patients seem to be more prone to poor outcome than are other critically ill patients due to both hyperglycaemia and hypoglycaemia [4–6]. Survival of trauma patients with out-of-hospital cardiac arrest is still low [7].

There is little data about the association between pre-hospital blood glucose concentration and dysrhythmias or cardiac arrest in trauma patients. The aim of this trial was to analyse the association between pre-hospital blood glucose concentrations and documented cardiac rhythms in trauma patients following arrival of the emergency physician. We particularly focused on the association between cardiac arrest and return of spontaneous circulation (ROSC) among pre-defined blood glucose levels. This information, in addition to vital parameters, could be helpful since measurement of blood glucose is simple, rapid, and inexpensive and may complement clinical assessment of patients at increased risk at the accident site.

The primary outcome of this study was the level of blood glucose observed during various cardiac rhythms in adult trauma patients. Secondary outcome parameter was blood glucose and its association with the rate of cardiac arrest and ROSC on scene. In addition, we also evaluated the predictive value of blood glucose in trauma patients who suffered cardiac arrest during emergency treatment.

## Methods

### Study design, inclusion and exclusion criteria

A retrospective analysis of data from pre-hospital missions conducted by the Helicopter Emergency Medicine Service (HEMS) of Allgemeiner Deutscher Automobil Club (ADAC) in Germany was performed. A nationwide, multicentre study including all 34 ADAC helicopter bases was conducted and all trauma patients treated by ADAC-HEMS between 1 January 2005 and 31 December 2013 were screened for inclusion. Inclusion criteria were adult trauma patients (≥18 years) treated by HEMS, in whom initial cardiac rhythms and blood glucose concentrations were documented. Exclusion criteria were interhospital transfers and incomplete or incongruent data recording (demographic data, cardiac rhythm, vital signs, injury pattern, trauma causes and courses). The study was approved by the Ethics Committee of the Medical Association of the Saarland and by the Institutional Review Board.

### Data processing

The following parameters were routinely recorded according to the predefined emergency physician dataset (Minimaler Notarzt-Datensatz, MIND2 [8]) within the observational database of the ADAC (LIKS® (Luftrettungs-, Informations- und Kommunikations-System)): demographic data, first vital parameters (heart rate, breathing frequency, systolic blood pressure) upon arrival of the professional rescuers, Glasgow Coma Scale (GCS) [9]), trauma mechanism, clinical evaluation of injury severity of the following body regions: head/brain, neck, face, chest, abdomen, thoracic and lumbar spine, pelvis, upper and lower extremities (1 = no injury, 2 = minor injury, 3 = moderate injury, 4 = severe injury, not life-threatening, 5 = severe injury, life-threatening, 6 = critical injury, life-threatening, 7 = deadly injury), whole injury pattern (1 = single injury, 2 = multiple injuries, 3 = polytrauma defined as life-threatening multiple trauma), the modified National Advisory Committee for Aeronautics (NACA) Index [10], 0 = no injury, 1 = minor injury, no intervention by a physician necessary; 2 = minor to moderate injury, ambulatory evaluation, 3 = moderate to severe injury, not life-threatening, in-patient care necessary, 4 = severe injury, potentially life-threatening, emergency physician care necessary, 5 = acute life-threatening injury, 6 = apnoea and circulatory arrest/resuscitation, 7 = deceased; of note: we included only patients who were alive on arrival of the HEMS emergency physician at the accident scene). In addition, the given volume, type of drugs administered, and rescue intervals were recorded.

### Blood glucose measurement

Blood glucose (in mmol/L) was measured at the scene with varying point-of-care devices that differed in accuracy and manageability. In most cases, glucose was measured from blood drawn immediately after venous access before any drug or volume administration. Blood glucose concentrations were categorised in groups: ≤2.80 mmol/L (50 mg/dL), 2.81–4.20 mmol/L (51–75 mg/dL), 4.21–5.55 mmol/L (76–100 mg/dl), 5.56–7.50 (101–135 mg/dL), 7.51–10.0 mmol/L (135–180 mg/dL), 10.01–15.0 mmol/L (181–270 mg/dL) and > 15.0 mmol/L (> 270 mg/dL). Hypoglycaemia and hyperglycaemia limits are not consistently defined to date and differ strongly in the literature. The thresholds of 2.80 (50 mg/dL), 4.2 mmol/L (75 mg/dL) and 5.55 mmol/L (100 mg/dL) are commonly used to define various stages of hypoglycaemia; whereas the threshold of 10 mmol/L (180 mg/dL, hyperglycaemia) is commonly used to define hyperglycaemia in pre-hospital emergency medicine and in-hospital intensive care medicine. The values between 5.56 mmol/L (> 100 mg/dL) and 7.50 mmol/L (135 mg/dL) are regarded as physiological blood glucose concentrations following normal nutritional intake; values exceeding 15 mmol/L (270 mg/dL) are defined as excessive hyperglycaemia [4, 5, 11–13].

### Cardiac rhythm analysis

Although automatic interpretation of some ECG findings is offered by most ECG devices, the binding diagnosis was performed by the emergency physician on service following immediate monitoring on site. Emergency physicians were trained during their practical year, of which four months were in internal medicine and another four months in anaesthesiology, during five years of specialisation (most of them in anaesthesiology and intensive care medicine), during their post-graduate training in emergency medicine (subspecialty qualification emergency medicine) including minimum 100 missions with ground EMS before applying for further training with HEMS. [14]. Routinely, a 3-lead ECG was established for initial rhythm diagnosis. In patients with signs of ischaemia an additional 12-lead ECG was written.

*Bradyarrhythmia* in adults was defined according to current guidelines as a heart rate ≤ 60 beats per minute [15]. Regular supraventricular bradycardia matches sinus bradycardia. Irregular supraventricular bradycardia included atrial fibrillation with slow ventricular response and sinus rhythms with relevant ventricular or supraventricular extrasystole. Ventricular bradycardia included ventricular escape rhythm, sinus arrest, sino-atrial exit block, high-grade second- and third-degree atrioventricular block, broad complex escape rhythm, and idioventricular rhythm.

*Tachyarrhythmia* in adults was defined as a heart rate > 100 beats per minute [15]. Regular tachycardia included sinus tachycardia, atrial tachycardia, paroxysmal supraventricular tachycardia, narrow-complex tachycardia, atrioventricular nodal re-entry tachycardia, sinus node re-entry, junctional tachycardia, Wolff-Parkinson-White syndrome. Irregular supraventricular tachycardia included focal atrial tachycardia, atrial fibrillation with rapid ventricular response as well as sinus tachycardia with relevant supraventricular and ventricular extrasystole. Ventricular tachycardia defined perfusing ventricular tachycardia.

*Normofrequent arrhythmia* included sinus rhythm with ventricular and supraventricular extra beats and irregular supraventricular arrhythmia with normofrequent ventricular response.

*Cardiac rhythms associated with cardiac arrest* were asystole, pulseless electrical activity (non-shockable rhythms), and ventricular fibrillation and pulseless ventricular tachycardia (shockable rhythms) [15]. Cardiac arrest was diagnosed according to ECG rhythm analysis (asystole, pulseless electrical activity (PEA), ventricular fibrillation or pulseless ventricular tachycardia), NACA score of 6 or 7, and documented as cardiopulmonary resuscitation. ROSC was measured when spontaneous circulation occurred during cardiopulmonary resuscitation on site. Successful cardiopulmonary resuscitation was defined by both a documented ROSC and a NACA score of 6 on admission.

### Statistical analysis

Statistical analysis was conducted with IBM SPSS Statistics (Release 24.0, 2016, Armonk, NY, USA). The Shapiro-Wilk test was used to test for normal distribution. Following descriptive analysis, the Mann-Whitney *U* test was used to compare group differences and the chi-square test was performed to detect frequency differences. For the prediction of cardiac arrest (NACA score 6 or 7) we applied a generalised additive model [16] using common vital parameters for model 1 (heart rate, respiratory frequency, systolic blood pressure, GCS) and for model 2 common vital parameters *and* blood glucose on site. More precisely, the effects of the predictor variables were modelled using penalised regression splines [17] to be able to identify potentially nonlinear relationships between cardiac arrest states with changing vital parameters. The models were estimated using the statistical environment R [18] and the recommended mgcv package [19]. Integrated discrimination improvement (IDI) and net reclassification improvement (NRI) were used to assess the improvement of outcome prediction comparing model 1 and model 2 (STATA/MP, release 13, College Station, TX, USA). Confidence intervals (CI) in this study were 99%. A *p* value of 0.01 was deemed to be statistically significant.

## Results

### Patient population

**Fig. 1:**
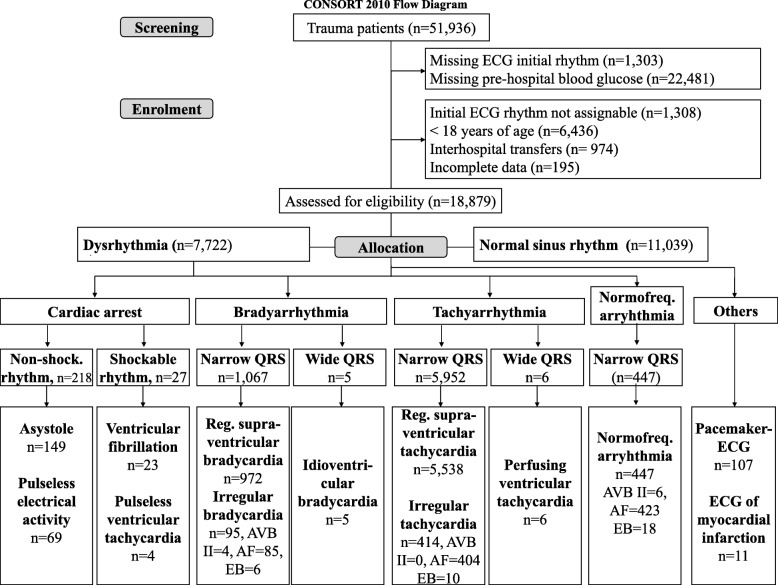
Consort 2010 Flow Diagram for screening, enrolment, allocation and analysis of trauma patients. ECG: Electrocardiogram, QRS: QRS complex of ECG analysis, AVB: atrioventricular blockage, AF: atrial fibrillation, EB: extra beats, namely supraventricular and ventricular extrasystole, Normofreq: normofrequent, Non-shock: non-shockable, Reg: regular

Of 51,936 trauma patients, 28,152 patients with recorded ECG findings *and* glucose concentrations were eligible; 18,879 trauma patients fulfilled the inclusion criteria and were enrolled (13,185 (69.8%) were male; mean age 50 ± 20 years). In 58.5% (11,039/18,879) of the trauma patients ECG findings were within normal limits, in 31.6% (5958/18,879) ECG showed tachycardia and 5.7% (1072/18,879) had bradycardia. Cardiac arrest was diagnosed in 466 (2.5%) of the trauma patients; 185 patients (1.0%) died on the scene (Fig. 1, Table 1), and 3064 (16.3%) patients had single injuries (predominantly severe to life-threatening head injuries), while 13,031 (69.0.1%) patients had multiple injuries, and 2784 (14.7%) patients were polytraumatised.

**Table 1:** Initial blood glucose levels in mmol/L, rate of cardiac arrest and return of spontaneous circulation (ROSC) during various initial cardiac rhythms observed in adult trauma patients (*n* = 18,879)

	Blood glucose	p	Age	p	Cardiac arrest in relation to initial glucose concentration			ROSC in relation to initial glucose concentration		
					≤4.20 mmol/L	4.21–10.0 mmol/L	> 10.0 mmol/L	≤4.20 mmol/L	4.21–10.0 mmol/L	> 10.0 mmol/L
Normal sinus rhythm	6.7 ± 2.1		50.9 ± 19.4		0/372 0%	56/10,080 0.6%	14/587 2.4%	–	43/56 76.8%	11/14 78.6%
Shockable rhythms										
Ventricular fibrillation Pulseless ventricular tachycardia	9.0 ± 3.2	< 0.0001	64.2 ± 12.9	0.0004	1/1100%	14/14100%	8/8100%	1/1100%	10/14 71.4%	7/8 87.5%
	9.8 ± 1.8	0.008	58.5 ± 12.5	0.44	0	2/2100%	2/2100%	–	2/2100%	2/2100%
Non-shockable rhythms										
Asystole Pulseless electrical activity	6.7 ± 2.4	0.69	48.8 ± 19.4	0.21	20/20100%	113/113100%	16/16100%	4/20 20%	40/113 35.4%	8/16 50%
	6.6 ± 2.3	0.45	57.2 ± 20.4	0.015	4/4100%	60/60100%	5/5100%	1/4 25%	32/60 53.3%	3/5 60%
Bradyarrhythmia	6.9 ± 2.4	0.036	58.0 ± 20.5	0.0004	3/50 6.0%	25/943 2.7%	4/79 5.1%	2/3 66.7%	17/22 77.3%	3/4 75%
Tachyarrhythmia	7.0 ± 2.4	< 0.0001	45.8 ± 19.3	< 0.0001	3/186 1.6%	88/5298 1.7%	17/475 3.6%	2/3 66.7%	70/88 79.5%	15/17 88.2%
Normofrequent Arrhythmia	8.0 ± 3.1	< 0.0001	77.3 ± 11.2	< 0.0001	0/9	1/359 0.3%	3/79 3.8%	–	1/1100%	3/3100%
Other										
Pacemaker ECG	8.1 ± 3.1	< 0.0001	76.5 ± 9.6	< 0.0001	–	1/87 1.1%	1/20 5.0%	–	1/1100%	1/1100%
Myocardial infarction ECG	7.2 ± 1.2	0.051	69.1 ± 15.4	0.0027	–	5/11 45.5%	–	–	3/5 60%	–

ROSC: return of spontaneous circulation; ECG: electrocardiogram, p in comparison to patients with normofrequent sinus rhythm

### Blood glucose and cardiac rhythms

Patients with tachycardia (≥100/min, 7.0 ± 2.4 mmol/L, *p* < 0.0001), pulseless ventricular tachycardia (9.8 ± 1.8, mmol/L, *p* = 0.008) and patients with ventricular fibrillation (9.0 ± 3.2 mmol/L, p < 0.0001) had higher blood glucose than did patients with normal sinus rhythm of 61–99/min (6.7 ± 2.1 mmol/L). Patients with asystole (6.7 ± 2.4 mmol/L) or pulseless electrical activity (PEA, 6.6 ± 2.3 mmol/L) and bradycardia (6.9 ± 2.4 mmol/L) had comparable blood glucose levels. (Table 1).

### Blood glucose and cardiac arrest

The frequency of patients with cardiac arrest was highest in patients with either hypoglycaemia (≤4.2 mmol/L; 31/641; 4.8%, ≤2.8 mmol/L; 7/79; 8.9%) or hyperglycaemia (> 10 mmol/L; 68/1270; 5.6%, > 15 mmol/L; 16/264; 6.1%) and lowest in patients with blood glucose of > 4.2–7.5 mmol/L (262/13,780; 1.9%). (Fig. 3) In 80% (174/218) of the patients with asystole or pulseless electrical activity a life-threatening polytrauma was diagnosed, whereas 20 (60.6%) of the 33 patients with ventricular fibrillation or ventricular tachycardia suffered from a single injury.

**Fig. 2:**
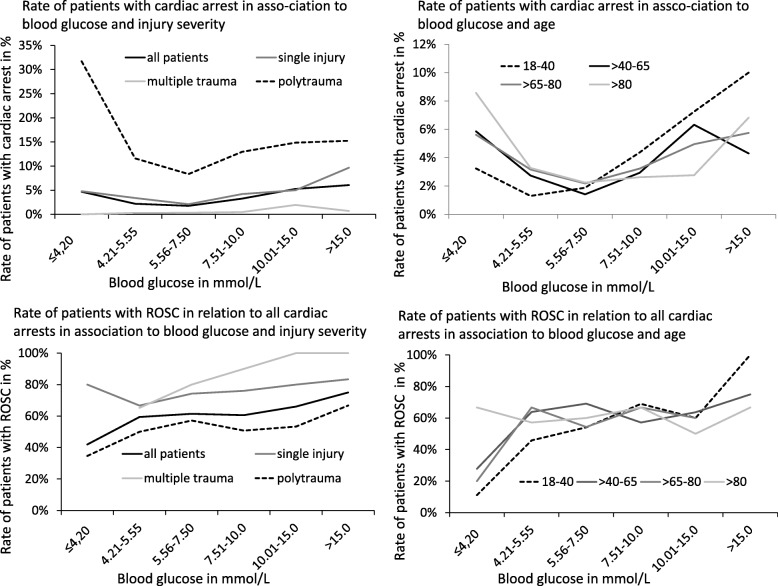
Number of patients with cardiac arrest and frequency of return of spontaneous circulation (ROSC=NACA 6) in association with initial blood glucose levels, injury pattern, and age. Small case numbers caused the hypoglycaemic categories of < 2.80 mmol/L and 2.81–4.20 mmol/L to be merged

Especially in polytraumatised patients, pre-hospital blood glucose showed a significantly U-shaped association with the rate of patients with cardiac arrest (*p* < 0.0001), with the lowest rate of cardiac arrest being in patients with blood glucose at 5.56–7.5 mmol/L (112/1340, 8.4%) and the highest rate in hypoglycaemic patients (≤4.2 mmol/L, 26/82, 31.7%). This U-shaped pattern was less marked in patients with a single injury and was not observed in patients with multiple injuries. (Fig. 2) This U shape was also found in all age categories (p < 0.0001). In patients ≤40 years the rate of cardiac arrest was higher with hyperglycaemia (> 10 mmol/L, 14/178, 7.9%; > 15 mmol/L, 4/40, 10.0%), whereas in patients > 40 years the rate of cardiac arrest was higher with blood glucose levels < 4.2 mmol/L (22/363, 6.1%).

**Fig. 3:**
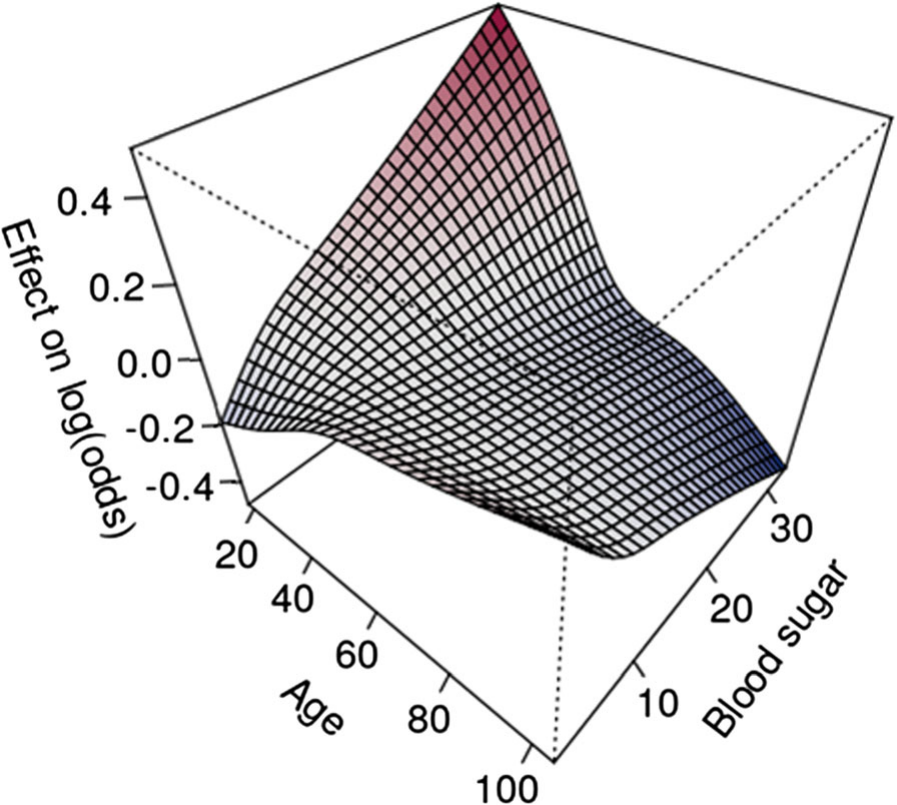
Estimated smooth interaction effect of age and blood glucose of the GAM model 2 (vital signs and blood glucose). The figure indicates an increased risk for NACA 6 or 7 to result in higher blood glucose values for young people and the inverse effect for people older than 40 years

Prevalence of dysrhythmias and cardiac arrest was related to age. Analysing age and blood glucose for their combined association to cardiac arrest revealed that young age <40 years and high blood glucose as well as age > 65 years and low blood glucose indicate an increased risk for cardiac arrest in all trauma patients. (Fig. 3) No significant differences were seen between mean peripheral oxygen saturation in bradycardia, tachycardia or in normofrequent rhythms.

In patients with minimal circulation (heart rate > 30/min and systolic blood pressure > 40 mmHg; *n* = 18,633) [20] on arrival of the emergency physician, pre-hospital blood glucose non-significantly improved the prediction of cardiac arrest (surrogate parameters NACA score 6 or 7, area under the curve 0.881 with common vital signs, 0.886 for common vital signs and blood glucose; IDI *p* = 0.03, NRI *p* = 0.68 in all patients) in comparison to prediction by common vital signs alone (heart rate, breathing frequency, Glasgow Coma Scale, blood pressure).

### Blood glucose and ROSC

Blood glucose was measured in 466 patients with pre-hospital traumatic cardiac arrests.

The frequency of ROSC (NACA score 6) in all patients with cardiac arrest (NACA score 6 or 7) increased with rising blood glucose: from 13/31 (41.9%) in patients with blood glucose ≤4.2 mmol/L, to 221/366 (60.4%) in patients with blood glucose of 4.21–10.0 mmol/L, to 47/69 (68.1%) in patients with blood glucose of > 10 mmol/L. The rate reached even 100% in younger patients (18–40 years) with excessive hyperglycaemia (> 15 mmol/L). (Fig. 3).

Only 43 (54%) of 79 patients with hypoglycaemia ≤2.8 mmol/L and 166 (26%) of 641 patients with hypoglycaemia ≤4.2 mmol/L received iv glucose therapy. In hypoglycaemic patients with cardiac arrest and documented iv glucose administration, there was a trend towards higher survival rate in comparison to hypoglycaemic patients without documented glucose administration: hypoglycaemia ≤4.20 mmol/L: cardiac arrest 31 patients, of whom six received iv glucose, four of them survived to hospital admission (ROSC) = 66.7%, 25 received no iv glucose, eight of them survived to hospital admission (ROSC) = 32%, *p* = 0.174.

## Discussion

In this retrospective analysis of 18,879 trauma patients we demonstrate that prehospital dysrhythmia was associated with significantly deranged blood glucose concentrations. Patients with cardiac arrest presented with blood glucose concentrations in a U-shaped manner. This was especially evident in polytraumatised patients ≤40 years with either hypoglycaemia (32%) or hyperglycaemia (15%). Furthermore, the rate of ROSC correlated positively with initial blood glucose levels.

In cardiac arrest patients with high-frequency rhythms such as tachycardia or ventricular fibrillation we observed significantly higher blood glucose levels than in patients with pulseless electrical activity and asystole. To put it another way, 77.4% of cardiac arrest patients with hypoglycaemia (≤4.2 mmol/L) presented with asystole or pulseless electrical activity, whereas only one patient presented with ventricular fibrillation. The heart relies primarily on augmented glucose utilisation to meet energetic needs for force generation. Increased heart work, usually elicited by catecholamines, increases carbohydrate oxidation because of activation of the pyruvate dehydrogenase complex [21]. Amazingly, administration of i.v. glucose was recorded in only half of the patients with severe hypoglycaemia and in only one-quarter of the patients with moderate hypoglycaemia.

Except in patients with diabetes mellitus, acute hyperglycaemia following trauma is mainly a consequence of distress causing a hypothalamic-hypophysic-adrenal stress response modulated by trauma severity, incidence of shock, and age [22–24]. Haemorrhagic shock and hypoxaemia belong to the strongest stressors in mammals, triggering highest levels of cortisol and catecholamines [24–26]. They lead to release of pro-inflammatory cytokines in the liver [27, 28], trigger glycogenolysis, and gluconeogenesis by degradation of muscle lactate, glucoplastic amino acids, and glycerol in liver and kidneys, and lipolysis [29–31]. Simultaneously, tumor necrosis factor α induces a peripheral insulin resistance [32]. This stress response-induced hyperglycaemia supports initial steps of immune defense and wound healing. In addition, hyperglycaemia leads to a higher concentration gradient to tissues with disturbed microcirculation and increased need, especially in the brain following injury [33–35], which eases glucose uptake. Over and above this, hyperglycaemia may improve cardiac function and resistance during stress and osmotic effects counteract blood loss [36–39].

In severely injured patients who were found to be hyperglycaemic on arrival of the emergency physician, circulation presumably lasted long enough to develop a stress response. In contrast, patients with asystole or pulseless electrical activity had less time for a physical stress response. This assumption is supported by the fact that 80% of the patients with asystole or pulseless electrical activity were polytraumatised, whereas patients with ventricular fibrillation or ventricular tachycardia had suffered a single injury in 60% of the cases in our study.

The potentially positive effects of hyperglycaemia in the acute post-traumatic situation are accompanied by negative sequelae from prolonged hyperglycaemia known as “diabetes of injury” [40, 41], which seems to be more pronounced than diabetes mellitus-induced hyperglycaemia. [42, 43].

The high frequency of hypoglycaemic patients in cardiac arrest needs further investigation. The prevalence of diabetes mellitus among adults in the German population averages about 7–8%, with increasing prevalence depending on age [44]. Theoretically, in some of the diabetic trauma patients hypoglycaemia may have been a consequence of anti-hyperglycaemic drug overdose from insulin or anti-diabetic drugs. In addition, hypoglycaemia in non-diabetic patients could have resulted from extensive shivering due to hypothermia, due to exposure to cold and wet environment, but also from chronic liver disease, intoxication, or severe liver and kidney trauma [22, 23, 45–48].

The finding that the rate of successful resuscitation attempts correlated with blood glucose levels, especially in polytraumatised and young patients, raises the question whether blood glucose levels need to be increased during CPR in patients with traumatic cardiac arrest. Some studies support the hypothesis that hyperglycaemia could be beneficial during cardiac arrest: Nehme et al. observed that diabetes affects at least one in five patients who have had an out-of-hospital cardiac arrest and is associated with poorer survival and 12-month functional recovery. In contrast, a mild-to-moderate elevation of pre-hospital blood glucose level was associated with improved survival and functional recovery, which were independent of diabetes status [49]. Mentzelopoulos found better outcome by administering – among others - blood glucose-increasing steroids for resuscitation of in-hospital cardiac arrest [50]. In animal studies, hyperglycaemia during cardiac arrest led to greater cerebral oxygenation [51], and blood glucose-increasing glucagon administration during cardiac arrest improved survival rate, cardiac function, and neurological outcome [52, 53]. Hyperglycaemia was associated with reduced myocardial infarction size and improved systolic function during myocardial ischaemia [37]. In traumatised patients and patients with sepsis, glucose uptake in macrophage-rich tissues is significantly increased [54]. A substantial hyperglycaemia level may overcome local or general microcirculation disturbances (injuries, sepsis, ischaemia) by increasing the concentration gradient, which facilitates non-insulin-dependent glucose uptake. These positive findings are accompanied by a long list of publications with negative results regarding hyperglycaemia and outcome in several diseases and critical conditions [2, 55–60]. Russo et al. retrospectively investigated clinical outcome in relation to mean blood glucose during the first 96 h after hospital admission in comatose survivors of out-of-hospital cardiac arrest with an initial shockable rhythm. They found that higher mean blood glucose levels during the first 96 h after admission were associated with increased rates of death and severe neurological dysfunction [61]. However, initial blood glucose level could be a surrogate marker of ischaemic insult severity during cardiac arrest [62].

After all, measuring blood glucose during pre-hospital care of trauma patients is easy, rapid, inexpensive and may yield additional information to estimate or complement clinical assessment of a patient’s pre-hospital situation as a whole.

### Limitations

Limitations of this study are its retrospective design, although all data were collected prospectively.

In our study about 46% of the trauma patients were excluded mostly due to missing prehospital glucose measurement or ECG rhythm documentation (Fig. 1). Thus, we cannot exclude selection bias, especially in the more severe cases in which HEMS physicians focus on supporting vital functions rather than on lab investigations. Patients in category NACA 7 were more numerous in the excluded population than in study patients.

In addition, we have no in-hospital data. In particular, we lack information on the frequencies of confirmed diagnoses and injury patterns, the in-hospital course of blood glucose concentrations and the ultimate outcome. However, this does not affect the core parameters of our study, initial ECG and on-site blood glucose concentrations. Worse, there is no on-site information available about pre-existing diseases such as diabetes, which probably influenced the course. The prevalence of diabetes in the German population is quoted as being 7–8% [44]. Accordingly, about 1500 patients in the study population may have been diagnosed with diabetes. We do not know the frequency of study patients with diabetes complicated by vascular and end-organ damage and we cannot tell how many of them were under anticoagulation therapy or had taken anti-diabetic drugs. Furthermore, our results regarding outcome of hypoglycaemic trauma patients do not consider administration of glucose in half of them. The extent to which oral anti-diabetic drugs or insulin may influence blood glucose concentrations during trauma and shock is not known and may vary individually with the time of drug ingestion/administration, the extent of oral carbohydrate intake, and the individual patient’s stress response. In recent studies it was reported that stress-induced hyperglycaemia rather than diabetic hyperglycaemia is associated with higher mortality in trauma [42, 43].

Another problem may arise from differences in point-of-care devices and with either venous or capillary blood measurements when haemodynamic shock developed. Routinely, blood glucose concentrations in pre-hospital trauma patients were measured from blood drawn from venous access before any drug or volume administration. However, we cannot exclude that in selected cases capillary blood glucose was measured by ear or finger sticks. The literature shows contradictory conclusions regarding the impact of venous vs. capillary blood glucose measurements, the existence of shock or the administration of catecholamines. In addition, the limited precision of point-of-care devices is well-known, especially when blood glucose concentrations are extremely high or low [63–65]. In this study, measurements of blood glucose concentration were conducted while establishing the initial iv access and before drug administration, for which reason the influence of external catecholamines (e.g. in the context of cardiopulmonary resuscitation) can be excluded as far as possible.

## Conclusions

In adult trauma patients, higher pre-hospital blood glucose levels were related to tachycardic and shockable rhythms. Cardiac arrest was more frequently observed in hypoglycaemic and hyperglycaemic pre-hospital trauma patients. The rate of ROSC rose significantly with initial blood glucose. Blood glucose measurements in addition to common vital parameters (GCS, heart rate, blood pressure, breathing frequency) may help identify patients at risk for cardiopulmonary arrest and dysrhythmias. Therefore, it may be prudent to routinely measure blood glucose concentration during initial emergency care in pre-hospital trauma patients.

## Abbreviations

ECG: electrocardiogram
GCS: Glasgow Coma Scale
HEMS: Helicopter Emergency Medicine Service
IDI: integrated discrimination improvement
NACA: National Advisory Committee for Aeronautics
NRI: net reclassification improvement
PEA: Pulseless electrical activity
ROSC: Return of spontaneous circulation
